# Emotion Processing for Arousal and Neutral Content in Alzheimer's Disease

**DOI:** 10.4061/2009/278615

**Published:** 2010-02-01

**Authors:** Corina Satler, Carlos Uribe, Carlos Conde, Sergio Leme Da-Silva, Carlos Tomaz

**Affiliations:** ^1^Laboratory of Neurosciences and Behavior, Department of Physiological Sciences, Institute of Biology, University of Brasilia, Brasilia-DF CEP 70910-900, Brazil; ^2^Faculty of Health Sciences, Industrial University of Santander, A.A. 678, Bucaramanga, Santander, Colombia; ^3^Institute of Psychology, University of Brasilia, Brasilia-DF CEP 70910-900, Brazil

## Abstract

*Objective*. To assess the ability of Alzheimer's disease (AD) patients to perceive emotional information and to assign subjective emotional rating scores to audiovisual presentations. *Materials and Methods*. 24 subjects (14 with AD, matched to controls for age and educational levels) were studied. After neuropsychological assessment, they watched a Neutral story and then a story with Emotional content. *Results*. Recall scores for both stories were significantly lower in AD (Neutral and Emotional: *P* = .001). CG assigned different emotional scores for each version of the test, *P* = .001, while ratings of AD did not differ, *P* = .32. Linear regression analyses determined the best predictors of emotional rating and recognition memory for each group among neuropsychological tests battery. *Conclusions*. AD patients show changes in emotional processing on declarative memory and a preserved ability to express emotions in face of arousal content. The present findings suggest that these impairments are due to general cognitive decline.

## 1. Introduction

Over the last few years, attention has been focused on the processing of emotion in elderly individuals with a variety of progressive neurological disorders, particularly dementia. 

Although the interest in the emotional aspects of Alzheimer's disease (AD) has increased over the last years, the existence of a deficit in the perception or expression of emotional conditions is still widely debated. Indeed conflicting evidence has been presented, stemming in part from methodological inadequacies, and in part from lack of consideration of a specific deficit hypothesis.

Regarding the emotion expression, several studies indicate that it is largely intact in AD [1]. Specifically, it was found that AD and normal controls provided similar subjective ratings when viewing emotion-eliciting images and that these ratings were in the expected directions on valence and arousal dimensions [2]. In addition, participants with AD rated their emotional experiences similarly to control participants but differed in emotion expressivity: they exhibited more negative facial expressions while viewing sad vignettes [3]. 

About the emotional memory enhancement effect, a body of data indicates a relatively intact enhancement effect for positive pictures [4], negative stories or film clips [5–8], and a real-life event [9–11]; whereas contrasting results demonstrating a marked impairment in the enhancement effect for positive pictures [12, 13], negative pictures [4, 12, 13], negative and positive words, negative sentences [12], and negative and positive neutral words [14].

A similar contrasting profile is presented by studies regarding the emotional recognition contents. Early studies have generally demonstrated impairment of emotional processing abilities [15, 16], whereas more ones have demonstrated a preserved ability to recognize facial emotions [17–23]. 

When the correlations between ability to recognize emotions and the degree of disease severity and also the type of neuropsychological dysfunction were analyzed, it has been shown that impairment in the emotional processing may be due to cognitive deficits, rather than deficits in emotion processing per se [19, 24–26]. 

Given that the divergent results regarding the capacity to recognize emotion in complex stimuli are poorly investigated, the goal of the present study was to clarify the discrepancy on literature regarding the ability to perceive and to attribute an emotional rating to stories with different emotional valences and arousal (a neutral story and an emotionally negative). Our working hypothesis was that AD patients would have worse emotion processing than control subjects and there would also be a correlation between the performances in the emotional memory test and neuropsychological screening tools.

## 2. Materials and Methods

### 2.1. Participants

Twenty-four subjects participated in the study. The control group (CG) comprised of 10 subjects, and 14 AD patients were matched for age, gender, and years of education.

All participants were examined by neurologists and a psychologist and were submitted to standard neuropsychological examinations to assess their cognitive function (described in later section). Diagnosis of AD was established according to the NINCDS-ADRDA criteria [27] and the dementia severities were determined as 1 or 2 (9 and 5 AD patients, respectively) by the Clinical Dementia Rating (CDR) [28]. Patients with AD had a history of cognitive decline and memory problems but showed normal consciousness and they lived with their families, requiring no special care. Regarding hearing/vision problems, none of the participants showed deficiencies that would impair their tests' performance and the degree of loss reported by AD patients was matched to those of the control group. Patients with other specific causes of dementia such as brain lesions, delirium, and depression were excluded.

All AD patients were recruited from the University Hospital of Brasília (HUB), Brasília; each of whom had given informed consent prior to participation—in accordance with the ethical guidelines for research with human subjects (196/96 CNS/MS resolution).

### 2.2. Neuropsychological Evaluation

All subjects were tested with a standard battery of formal neuropsychological tests to evaluate the mental cognitive condition and severity of their cognitive impairment. For this study was used a Brazilian version of the Mini-Mental State Examination (MMSE), with cutoffs according to education level [29], to assess the cognitive mental state (Total score) and the person's orientation to time and place, recall ability, short-term memory, attention and arithmetic ability, and language comprehension and expression (subtests scores). The Clock Design (CLOX) [30] was used only in the first part (patients were asked to draw a clock and put 13:45 h) verifying executive control and semantic knowledge. Verbal Fluency test, FAS (oral fluency by letters F, A, and S), was used to assess inhibitory control, thought organization, speed of processing, and language. Semantic memory was evaluated by Category test (animals/min) [31]. The Five-Point Test [32] was used to evaluate cognitive flexibility and nonverbal fluency. To evaluate episodic memory (immediate and delayed), Logical Memory I and II of Wechsler Memory Scale Third Edition (WMS-III) was used. Other subtests, such as Digit Span (Forward and Backward), were used to assess short-term memory and working memory, along with Mental Control subtest attention and concentration functions. The Similarities subtest of Wechsler Adults Intelligence Scale-III (WAIS-III) evaluated the subject's ability to mentally process verbal information, categorizing, and conceptualizing information in the long-term memory store (abstract reasoning). Depressive symptoms were measured using the Geriatric Depression Scale (DGS) adapted to Portuguese [33].

Although the AD patients were not formally tested for propositional comprehension, they were able to interact and showed adequate understanding of the instructions during the neuropsychological interview.

### 2.3. Assessment of Emotion Processing

The instrument used was the Emotional Memory Test (EMT) [34] which consists of two stories. One was composed of relatively emotionally neutral film clips (Neutral), and the other comprised relatively emotionally arousing film clips (Emotional). The test relies on the retention of information from an arousing experience that lacks traumatic intensity and involves visual and verbal modalities (see [35, 36]).

### 2.4. Procedure

We used stimuli and procedure identical to those published by Frank and Tomaz [36]. Subjects were initially submitted individually to a screening interview and neuropsychological examination. In the first session, each participant watched the test stimuli (Neutral version) and immediately afterwards was asked to rate the story in a scale of 1 to 4, with 1 indicating “not emotional” and 4 “highly emotional.” Five minutes later, the subjects were given an 11-item recall test. The photographs were presented one by one, and in the same order; meanwhile, the recognition questionnaire was applied to assess subjects' memory of story line. Two weeks later, they did the second part of the research. On this occasion, they watched the Emotional version and then were asked to rate the emotional content of the whole story. Both emotional rating and recognition questionnaire were also performed with this version of the test. We did not separate the groups into two subgroups due to the fact that all participants watched the neutral story in the first session and the emotional story in the second. In this way, there was no emotional influence that could possibly interfere with their performance.

Within-groups design (repeated measure) was chosen in order to maximize our sample size. We decide not to counterbalance the presentation order of the two versions of the test with the aim of preventing any emotional influence over the neutral story.

### 2.5. Statistical Analysis

Demographic characteristics were assessed by a *t*-test. The Kolmogorov-Smirnov test was used in order to assess the normal distribution of the dataset. 

Emotional rating scores and total answers for each version of the test were evaluated by a mixed-model ANOVA 2 × 2, with Version (emotional and neutral) as repeated measure within groups and Group (CG or AD) as between group's factors.

Possible differences in CDR 1 and 2 between recognition questionnaire (Neutral and Emotional version) and Emotional rating scores were evaluated by an independent samples *t*-test.

Stepwise linear regression analyses were performed for each group to determine the best predictors (from all neuropsychological test results) of four dependent variables of interest: Total score in the recognition questionnaire (Neutral and Emotional version) and Emotional rating (Neutral and Emotional version). 

Significance value was set at *P* < .05 for all the tests. All statistic analyses were performed using SPSS version 13.0 for Windows (2004).

## 3. Results

### 3.1. Demographic Characteristics

Table 1 presents demographic information and test scores. The CG subjects and AD patients did not differ significantly in age (*t*(24) *P* = .466) and education (*t*(24) *P* = .771). There was no evidence of depression in any subject according to Geriatric Depression Scale, considering a cutoff score of 6. The CG and AD groups did not differ significantly in GDS scores (*t*(22) = − 1.02, *P* = .32).

### 3.2. Emotional Memory Test

#### 3.2.1. Total Scores of Multiple Choice Recognition Questionnaires

The total scores on the Neutral version of the questionnaire were CG: 22.6 ± 1.57 (SD), AD: 16.92 ± 4.56 (SD) and for the Emotional version CG: 22.7 ± 1.16 (SD), AD: 17.78 ± 4.30 (SD). There was a statistically significant effect of Group, F(1, 22) = 14.00, *P* = .001, over the total score of the multiple choice recognition questionnaire. CG scored higher in both versions of the test. Neither Version, F(1, 22) = 1.82, *P* = .19 nor Group × Version interaction effect was observed, F(1, 22) = 1,14, *P* = .30. As expected, mean scores of the recognition questionnaire for the neutral version were different between CDR1 (M = 19.33, SD = 2.74) and CDR2 (M = 12.6, SD = 4.04), *t*(12) = 3.74, *P* = .003. Mean score of the recognition questionnaire for the emotional version was also different between CDR1 (M = 19.67, SD = 3.08) and CDR2 (M = 14.4, SD = 4.34), *t*(12) = 2.66, *P* = .021.

#### 3.2.2. Rating of the Emotional Charge of the Stories

**Figure 1:**
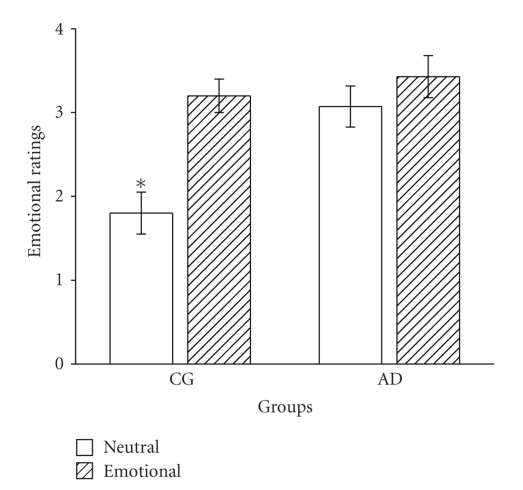
Ratings (mean ± SEM) of emotionality for neutral and emotional stories by the two groups: CG, control group, and AD, Alzheimer's patients. The subjects were asked to rate their emotional reaction to the slide show immediately after its presentation on a scale of 0 (not emotional) to 4 (highly emotional). *P < .005 was compared to AD for Neutral version (independent samples *t-*test). The scores assigned to the Emotional version were not different between the groups (*P* = .511).

Mean emotional ratings for Neutral version were CG: 1.80 ± 0.79 (SD), AD: 3.07 ± 0.92 (SD) and for the Emotional version: CG: 3.2 ± 0.63 (SD), AD: 3.43 ± 0.94(SD) (Figure 1). Emotional rating scores showed a significant effect of Group, F(1, 22) = 8.62, *P* = .008. In general, AD ratings were higher. A statistically significant effect of Version was observed too, F(1, 22) = 13.38, *P* = .001, as Emotional version of the story was rated higher than that of the Neutral one. And finally, a significant interaction Group × Version was found, F(1, 22) = 4.71, *P* = .04. Post hoc analyses revealed that CG assigned different scores for each version of the test, *t*(9) = −4.58, *P* = .001, while ratings of AD did not differ, *t*(13) = −1.05, *P* = .32.

No differences between CDR1 and CDR2 were observed in the emotional rating of any version (Neutral version: CDR1: M = 2.89, SD = 0.6; CDR2: M = 3.4, SD = 1.34; *t*(12) = −1.0, *P* = .34. Emotional version: CDR1: M = 3.78, SD = 0.44; CDR2: M = 2.8, SD = 1.3; *t*(12) = 2.10, *P* = .17).

#### 3.2.3. Linear Regression Analyses

The stepwise regression model found several predictors for the selected variables. Results are summarized in Table 2. CLOX test result was correlated to the Total score in both versions of the EMT for the AD group. In addition, WMS-R Mental Control result was included in the model to predict the Total score of the Neutral version. For the control group, WAIS-III Similarities test result predicted the Total score for the Emotional version of the EMT. Meanwhile, none of the neuropsychological test's results was a predictor for the Total score of the EMT's Neutral version for this group.

Regression analyses for the Emotional ratings found that the Neutral version rating was correlated to the result found in WAIS-III Similarities for the control group. For the AD group, MMSE and CLOX results were statistically correlated to the Emotional rating of this version of the EMT. For the Emotional version of the EMT, Verbal Fluency, Category (animals), was a predictor of the Emotional rating in the control group. Finally, Verbal Fluency, Letter (FAS), and MMSE-Recall results were found as predictors of the Emotional rating of this version in the AD group.

## 4. Discussion

In this study of emotion processing, the results are consistent with our hypothesis that AD patients would have a marked impairment in memory enhancement effect for EMT than that for CG subjects. Recall scores for both stories were significantly lower in AD patients than those in CG. As expected, AD patients classified at moderate stage of the disease (CDR 2) presented lower scores in the memory recognition questionnaire, in both versions, when compared to mild AD patients (CDR 1). The expected memory enhancement effect was not evident in the CG. This finding could be explained by a ceiling effect for the CG. However, in AD, there was no emotion enhancement of memory. These results regarding AD patients' performance in emotion tasks, specially in the memory recognition questionnaire, are different from those reported in recent studies using the same test [6, 7].

Analysis of each group individually (CG and AD) showed that, in CG, there was association with abstract reasoning (WAIS-III Similarities subtest) in the Emotional version. For AD patients, significant correlations were found, in both versions, involving specific functions such as auditory language skills, semantic knowledge, and executive functioning (CLOX) for both versions, and with attention and concentration functions too (WMS-R—Mental Control subtest) to Neutral version. Hence, it seems that AD patients had to turn to cognitive global functions to complete the task. Interestingly, they required more attention and concentration functions to Neutral version, suggesting that these materials come to distract them more than arousal contents.

On the other hand, given that EMT is a test made up of slide presentations of two short stories accompanied by a narrative, the present data support the hypotheses of several cognitive functions being engaged to undertake a complex task. In other words, the adequate global cognitive functioning and conservation of logical reasoning, apart from the mnemonic, linguistic processes (audio perception of stimuli, comprehension, and verbal understanding) and executive ones, seems crucial for a good performance in the test.

Regarding the attribution of emotion to the stories (Neutral and Emotional), AD subjects were able to recognize and assign appropriate emotion label associated with arousal content: it yielded no significant difference to CG. These results support the notion of preserved emotional content processing in AD patients. Despite methodological differences, these findings are in agreement with those from previous investigations [5, 17, 19, 20, 24–26] which support the idea of unimpaired emotional processing in AD, along with intact emotional expression [1–3, 37]. However, results for the Neutral version showed profound differences between the groups, in which CG subjects reported lower scores, as expected whereas AD patients gave similar values to the two versions.

Comparison between mild and moderate AD patients (CDR 1 and 2) shows no differences regarding emotional attribution to both stories. 

Focusing on the correlations between the attribution of emotion in EMT and neuropsychological test's scores, results suggest that attribution of emotion in its absence (Neutral version) involved for AD patients cognitive global functions and other specific domains such as semantic memory and executive control (MMSE and CLOX). For CG, there was a significant correlation with abstract verbal reasoning (WAIS-III Similarities subtest).

On the other hand, Emotional version involved, for both groups, specifically semantic memory (CG: Category test, animals/min and AD: Verbal Fluency, FAS), and additionally there was a significant correlation with other cognitive processes such as working memory, inhibitory control, thought organization, speed of processing, and language particularly for AD group (MMSE Recall subtest and Verbal Fluency, FAS). Therefore, whereas CG seemed to resort to mainly mnemonic processes, AD patients seemed to engage in a wide range of cognitive functions to complete the task, as suggested by the correlations analysis.

## 5. Conclusions

Our data regarding the emotional memory process indicate that AD patients did not benefit from emotional content cues. They maintained the ability to perceive and express emotions for arousal content but they were not as proficient in acknowledging emotions (or the lack thereof) for the neutral stimuli. In the context of AD, these difficulties are foreseeable since they are cognitively demanding and AD is, by definition, associated with prominent neurocognitive impairment. Thus, difficulties with neutral content may explain the different profile found between the groups.

The small sample size is a shortcoming that limits this study, and any conclusion should be interpreted with caution. Therefore, further studies, including larger samples of controls and AD subjects, as well as other neuropsychological tests are necessary to assess the influence of neutral and emotional processing in the early stage of AD.

## Figures and Tables

**Table 1:** Mean (±S.D.) demographic profiles of the subject populations.

Group	Age (years)	Gender	Education (years)	GDS
CG (*n* = 10)	70.3 ± 6.2	6 M, 4 F	8.1 ± 4.1	2.000 ± 1.76

AD (*n* = 14)	75.6 ± 5.3	6 M, 8 F	6.3 ± 3.2	2.71 ± 1.64

CG, control group; AD, Alzheimer's disease patients; GDS, Geriatric Depression Scale.

**Table 2:** Stepwise regression analyses results. SE, beta coefficient Standard Error; df, degrees of freedom; CG, control group; AD, Alzheimer's disease patients; CLOX, Clock Design Test; WMS-R, Wechsler Memory Scale-Revised; WAIS-III, Wechsler Adult Intelligence Scale-III; MMSE, Mini-Mental State Exam.

		b	SE	*t*	*P* value	Adjusted *R* ^2^	F	df	*P* value
*Total score*—*Neutral Version *									
AD									
	Constant	5.86	2.12	2.76	.018	*0.766 *	22.24	2, 13	<.001
	CLOX	1.11	0.22	5.17	<.001				
	WMS-R Mental Control	1.11	0.44	2.52	.029				
*Total score*—*Emotional Version *									
CG									
	Constant	20.16	0.76	26.44	<.001	*0.557 *	12.33	1, 9	.008
	WAIS-III Similarities	0.14	0.03	3.51	.008				
AD									
	Constant	11.90	1.60	7.42	<.001	*0.558 *	17.42	1, 13	.001
	CLOX	1.11	0.27	4.17	.001				
*Emotional rating*—*Neutral Version *									
CG									
	Constant	3.49	0.54	6.51	<.001	*0.528 *	11.06	1, 9	.010
	WAIS-III Similarities	−0.09	0.03	−3.33	.011				
AD									
	Constant	5.45	0.91	5.99	<.001	*0.545 *	8.78	2, 13	.005
	MMSE	−0.21	0.05	−4.12	.002				
	CLOX	0.24	0.08	2.81	.017				
*Emotional rating*—*Emotional Version *									
CG									
	Constant	5.12	0.63	8.11	<.001	*0.492 *	9.73	1, 9	.014
	Verbal Fluency—Category (animals)	−0.14	0.05	−3.12	.014				
AD									
	Constant	2.06	0.38	5.45	.002	*0.678 *	14.72	2, 13	.001
	Verbal Fluency—Letter (FAS)	0.10	0.02	4.58	.001				
	MMSE—Recall	−0.58	0.20	−2.94	.013				
